# Chemical and Physical Defense Traits in Two Sexual Forms of *Opuntia robusta* in Central Eastern Mexico

**DOI:** 10.1371/journal.pone.0089535

**Published:** 2014-03-05

**Authors:** Mariusz Krzysztof Janczur, Héctor Javier León Solano, Lupita Tzenyatze Solache Rámos, Citlalli Hypatia Mendoza Reyes, María del Carmen Oro Cerro, María Dolores Mariezcurrena Berasain, Irma Victoria Rivas Manzano, Javier Manjarrez, José Luis Villareal Benitez, Marcin Czarnoleski

**Affiliations:** 1 Research Group in Behavioral Biology and Conservation, Autonomous University of the State of Mexico, Toluca, State of Mexico, Mexico, Carretera Toluca-Tlachaloya, Toluca, Estado de México, México; 2 Research Group in Flora Composition, and Ecology of Tropical and Template Systems, Autonomous University of the State of Mexico, Toluca, State of Mexico, Mexico, Carretera Toluca-Tlachaloya, Toluca, Estado de México, México; 3 Faculty of Agricultural Sciences, Autonomous University of the State of Mexico, Toluca, State of Mexico, Mexico, Carretera Toluca-Tlachaloya, Toluca, Estado de México, México; 4 Research Group in Life History Evolution, Institute of Environmental Sciences, Jagiellonian University, Kraków, Poland; 5 Division of Computing Service, Information and Communication Technology, National Autonomous University of Mexico, Ciudad Universitaria, Mexico Distrito Federal, Mexico; Centro de Investigación y de Estudios Avanzados, Mexico

## Abstract

Sexually dimorphic plants provide an excellent opportunity for examining the differences in the extent of their defense against herbivores because they exhibit sex-related differences in reproductive investment. Such differences enable comparison of the sex with high reproduction expenses with the sex that expends less. The more costly sex is usually also better defended against herbivores. Generally, females are considered more valuable than hermaphrodites in terms of fitness; however, hermaphrodites are more valuable if they can produce seed by autonomous selfing, provided that the inbreeding depression is low and pollen is limited. We studied a gynodioecious population of *Opuntia robusta* from Central-Eastern Mexico, which has been reported to be trioecious, dioecious, or hermaphrodite, and addressed the following questions: 1) Is the hermaphrodite's reproductive output higher than the female's, and are hermaphrodites thus better defended? 2) Are plant tissues differentially defended? 3) Do trade-offs exist among different physical defense traits? and 4) among physical and chemical defense traits? We found that 1) hermaphrodites had a higher seed output and more spines per areola than females and that their spines contained less moisture. Non-reproductive hermaphrodite cladodes contained more total phenolic compounds (TPCs) than female ones. In addition, 2) hermaphrodite reproductive cladodes bore more spines than female cladodes, and 3) and 4) we found a negative relationship between spine number per areola and areola number per cladode and a positive relationship between spine number per areola per plant and TPC concentration per plant. Non-reproductive hermaphrodite cladodes contained a higher concentration of TPCs than female cladodes, and parental cladodes contained fewer TPCs than both reproductive and empty cladodes.

## Introduction

Differences in reproductive costs between sexual forms offer a unique opportunity to study resource allocation to competing functions. For example, females of woody plants typically use a higher fraction of available resources for reproduction than do males [1]–[3]. Eckhart & Seger [4] suggested that such sex-related differences in sexual reproduction should affect plant growth, phenology, floral and foliage traits, and tissue concentration of nutrients and anti-herbivore defenses, which should impose cascading effects on consumption rates of primary production at ecosystem levels. Indeed, evidence shows that herbivores typically feed preferentially on the biomass produced by male plants [5], [6], which suggests that female plants might spend more resources for defenses against herbivory to protect their investments in sexual reproduction. Also, it has been pointed out that herbivores may play a role in the divergence of female and male reproductive functions into separate individuals because leaf removal during flower development more negatively affects the male function of hermaphrodites, e.g., via reduction in pollen-tube growth rate [7]. Even when gynodioecy is supposed to be an intermediate stage in the evolution from hermaphroditism to dioecy [8], a process that sometimes is probably herbivore mediated [7], very little is known about plant–herbivore interactions of gynodioecious species [9].

It is not clear whether hermaphrodites invest more energy in reproduction than females or vice versa; however, hermaphrodites are expected to outperform females if they can produce seed by autonomous selfing provided that the inbreeding depression is low, and if pollen is limited. The latter condition occurs in habitats where many species share common pollinators and have similar flowering times, and a species of interest is not dominant [10], [11].

If one sexual form or one specific tissue is more valuable than others because more energy was invested in it, bringing about a differential allocation to the production of chemical defenses, a question arises: How is it possible that the most costly sex or organs were better defended if less energy was already left for defense? The explanation is that reproduction does not take energy from defense, but rather energy is allocated to the defense of a specific tissue because the reduction of fitness arising from its loss is greater than a lower allocation to its defense [12]. For this reason, we believe that the optimal defense theory (ODT) is concordant with the optimal energy allocation approach.

When defense is costly, it should be allocated to different plant tissues as a function of i) the rate of attack of a given tissue in the absence of defense; ii) the cost of employing the defense in that tissue; and iii) the value of this tissue for the plant or the cost of removing it [13]. Most recent studies also confirm some ODT predictions. For example, a meta-analysis carried out by McCall & Fordyce [14] confirmed that younger leaves have a higher concentration of defensive substances than older leaves; however, these authors did not find evidence that flowers were more defended than leaves. Furthermore, a meta-analysis study identified a positive (but non-significant) correlation between chemical and physical defenses [15].

If one of the two sexual forms of a gynodioecious population invests less energy in reproduction and at the same time is more damaged by herbivores, why do these sexual forms coexist? A few hypotheses can be put forward: 1) The population is in transition from gynodioecy to dioecy. In such a situation, one morph will be better defended and have the greater fitness. After an invasion of hermaphrodites by females, an invasion of a gynodioecious population by males and further extinction of hermaphrodites is expected [16]. 2) The more damaged sex compensates for the lack of resistance with tolerance, and the fitness of both morphs thus is similar. 3) A higher physiological cost of defense and reproduction of one sexual form is traded off by a lower survival, and a lower actual fitness in a less defended sexual form is thus compensated by a greater lifetime fitness. 4) The ecological cost of defense of each sexual form changes from season to season in such a way that in one season, one sex outcompetes another in terms of fitness, but the reverse is the case in another season; thus, the lifetime reproductive fitness of both forms is similar.

To test the ODT and its possible link with sexual polymorphism, we chose *Opuntia robusta*, a sexually dimorphic platyopuntia endemic to Mexico, with an ecology and life history that are almost unknown. Within its distribution zone, three kinds of populations have been reported: 1) exclusively hermaphroditic, 2) dioecious (males and females), and 3) trioecious (all three sexual forms). However, we found a non-reported population type, one that is gynodioecious. Previous studies suggest that in trioecious populations of this species, female individuals constitute 11% [17]. In this study, females accounted for 22.4% within 76 plants, probably suggesting that we found a population with a different sex proportion from those previously identified.

Because this plant presents not only chemical defense but also spines, we were able to test for a differential allocation to both resistance traits, either sex- or tissue-biased. Here we addressed the following questions: 1) Is the hermaphrodite's reproductive output higher than that of the female, and are hermaphrodites thus better defended? 2) Are plant tissues differentially defended? 3) Do trade-offs exist among different physical defense traits and 4) among physical and chemical defense traits?

## Materials and Methods

### Ethics statement

The research did not involve measurements on humans or animals. We obtained the permission of the head of the Municipality of Singuilucan, State of Hidalgo, Mexico (Secretario General Municipal de Singuilucan, Estado de Hidalgo, México) to carry out research activities on the lands administered by the Municipality: We acknowledge the administration of the Municipality for having given us permission to conduct the study on its territory. The owners of the land gave us permission to conduct the study on this site and were informed about the permission from the Municipality: We acknowledge the Pérez Juarez family for having allowed us to work on their land in San Nicolas Tecoaco. The study site is not considered a protected area [18], and *O. robusta* is not considered an endangered species [19]. To the best of our knowledge, during the study, we did not affect or involve any endangered species. No plant was killed or severely damaged as a result of our research activity; the plant material used for this study was sampled only at a very limited scale, and sampling therefore had negligible effects on broader ecosystem functioning.

### Study species

Our field study was conducted on *Opunta robusta* (Cactaceae), an endemic plant from the Meridional Altiplano, Mexico [20] (File S1).

Almost all mortality of the cladodes is caused by bugs from the *Chelinidea* genus (Coreidae; they eat the apical part of the young cladodes) and Hemiptera from the genus *Dactylopius* sp. (Dactylopiidae). In adult cladodes, the perforations made by the bugs cause necrosis and infection by pathogens [21]. In our study site, virtually all plants displayed either perforations or necroses produced by *Chelinidea*. The main mammal herbivores in the study area were the *Neotoma albigula* rat, goats (main vertebrate herbivore), horse, and donkey. Additionally, we observed *Sylvilagus* spp. rabbits, but we were unable to conclude whether they feed on *Opuntia*.

**Figure 1:**
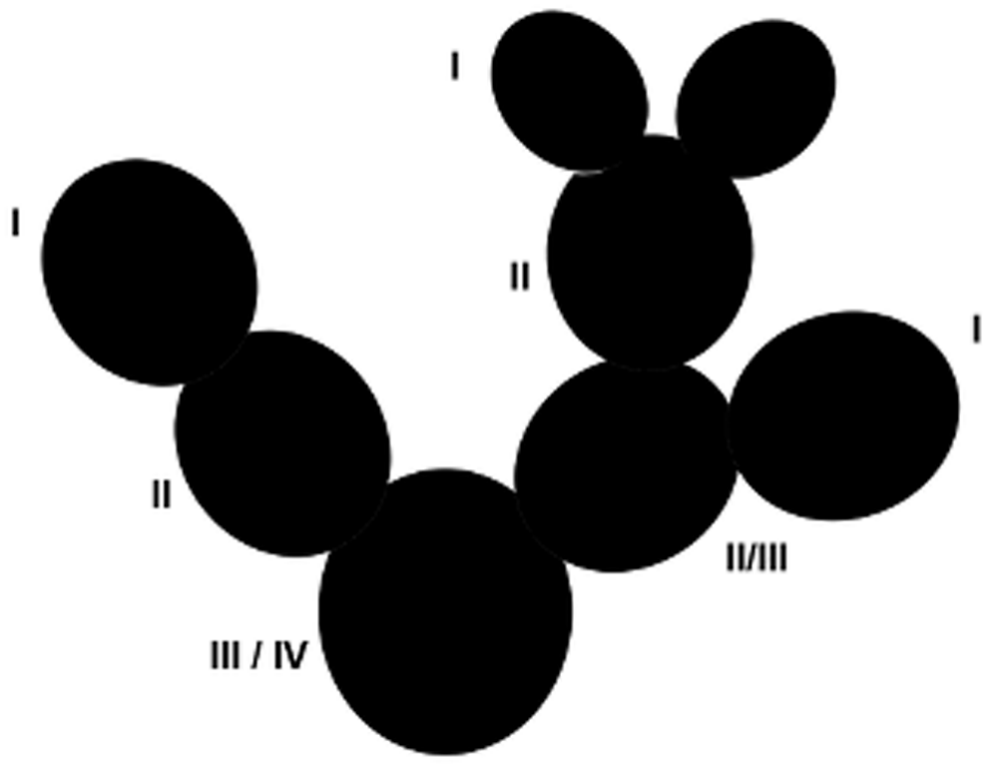
Schematic representation of *Opuntia robusta*. Cladodes of *O. robust*a are almost circular. Roman numbers describe the cladode levels. We call “1^st^-level cladodes” the newest ones (I). The oldest are called “4^th^-level cladodes” (IV in the figure). No plant in our sample had 5^th^-level cladodes. A 1^st^-level cladode that produces either one or two daughter cladodes (it never produces more than two) converts to a 2^nd^-level cladode. Daughter cladodes may appear on the same parental cladode in different seasons. In the same season, on the same plant, new cladodes can be daughters to the cladodes from different levels.

Figure 1 shows the schematic architecture of *O. robusta* and the nomenclature of cladode level we use here. The number of first-level cladodes can be as small as three and as high as 82 (we had only one plant from groups of these sizes, respectively).

### Study site

The study was carried out in Sierra de Pachuca, the State of Hidalgo (San Nicolas Tecoaco, Municipality of Singuilucan; 20 2′ 38.2″N, 98 35′ 16″W), an area of crassicaule scrubland with predominant *Opuntia* spp.; platyopuntias, barrel cacti, and agave species are the most conspicuous elements. The fieldwork was performed between February and December 2011. *O. robusta* accounted for approximately 40% of all *Opuntia* species in this zone.

### Plant choice in the study area

In the study site, we delimited an area of 300×150 m and randomly generated geographical positions of 300 points within this area, using the programming language Lazarus. We included plants situated within 5 m from these points. We chose 104 plants and numbered them with a permanent marker on the surface of chosen cladodes. During the blossoming period, we determined their sex. We consider only those plants that blossomed during the study.

### Sex determination

To determine the sex of the plants, we followed [10], [11], and [17]: white, empty anthers, short style and well-developed, lobular stigma characterize female flowers, and longer-than-female style and functional anthers characterize hermaphrodite individuals (Figure S1). We did not find male individuals. In previous studies, sex change has never been detected [10], [17].

### Plant architecture and size estimators

Similar architectures of both sexual forms would mean that plants from both groups likely had a similar age distribution; that is, we sampled from comparable plants. We considered the relationship between the numbers of cladodes of each level as a good estimator of plant architecture. We counted the number of cladodes from each level (1–4) on each plant; estimated the relationships between the ln-transformed numbers of the first and second levels, first and third levels, first and fourth, second and third cladodes, etc., for each sexual form; and compared both the slopes and the intercepts between sexes for all the combinations of cladode levels. Equal regression parameters for these relationships indicated that the architecture of both sexual forms is similar. On the other hand, if inter-sexual differences in architecture had emerged, we would have had to consider them as an outcome of possible sex-related differences in sexual reproduction that affected growth and growth-related traits.

To test whether possible sex-related differences in sexual reproduction affect other growth-related traits, we used different measures to compare plant sizes between sexual forms: 1) the number of cladodes from each level (Kruskal-Wallis or, K-W); 2) plant extensions (length, width, and height; ANOVA), by measuring maximum plant length and width parallel to the soil and maximum height in the center of the plant, using measuring tape; and 3) first-level cladode length and width taken with a measuring tape in August 2011, during the rainy season. Because of the field-work load, we were unable to make this measurement for all plants and did so for only 13 females (372 cladodes) and 30 hermaphrodites (697 cladodes). We compared these values using a GLM (General Linear Model), treating the variable “plant” nested inside the variable “sex” as a random factor and the variable “cladode” as a repetition [22]. If a cladode shared levels (e.g., 2 and 3; Figure 1), we assumed the higher (older?) level.

### Selection of cladodes for the comparison of physical and chemical defense traits

We designated as “parental cladodes” those that produced new-growing cladodes during the study season; as “reproductive cladodes” those that reproduced in the same season; and as “empty cladodes” those that were neither reproductive nor parental. We sampled independently cladodes for the estimation of physical and chemical defense.

To separate the effects of the different cladode states on the defense traits, we assigned the plants to the following groups: E, plants with empty cladodes only; PE, plants with parental and empty cladodes; RE, plants with reproductive and empty cladodes; and RPE, plants with each cladode in a different state (reproductive, parental, and empty). Simultaneous parental and reproductive cladodes were very infrequent. We refer here only to the state of the three sampled cladodes; e.g., the group name “plants with empty cladodes” does not mean that a plant lacked reproductive or parental cladodes but rather that only empty cladodes were chosen during random sampling from a given plant. Cladodes for the determination of physical and chemical traits were chosen independently [23].

We used the following number of cladodes per group for the determination of physical defense traits: E, 21 vs. 78; PE, 6 vs. 9; and RE, 21 vs. 84, for female and hermaphrodite cladodes, respectively. Group RPE contained two individuals and was not analyzed. For the determination of total phenolic compounds (TPCs), the cladodes and plants were assigned as follows (number of individuals in parentheses): E, 9 (3) vs. 27 (9); PE, 14 (5) (one tissue sample was lost) vs. 28 (10) (two tissue samples were lost); RE, 6 (2) vs. 62 (21) (one tissue sample was lost); and RPE, 18 (6) vs. 32 (11) (one tissue sample was lost), for female and hermaphrodite, respectively. When no sample was lost, the number of cladodes corresponds to three per plant. We compared defense traits according to the cladode state composition in a group; e.g., for group E, we compared the average value of a trait per cladode, treating cladodes of the same plant as repetition; for group PE, parental state and sexual form of the cladodes as well as the interaction between these two states were compared, treating the average value of the trait on a cladode as a repetition, etc. We used the GLM with the variable “plant” as a random factor nested in the variable “sex” [22].

### State-dependent physical and chemical defense traits

We took samples of the total phenolic compounds at the end of the reproductive season (December 8 and 9, 2011): When the defense is not complete (generally the case with tannins), a higher concentration of the TPCs earlier in the vegetative season is also reflected in their higher concentration after maturation [12], [24], [25]. Additionally, during this stage, no fluctuations in TPC concentration occur [12].

**Figure 2:**
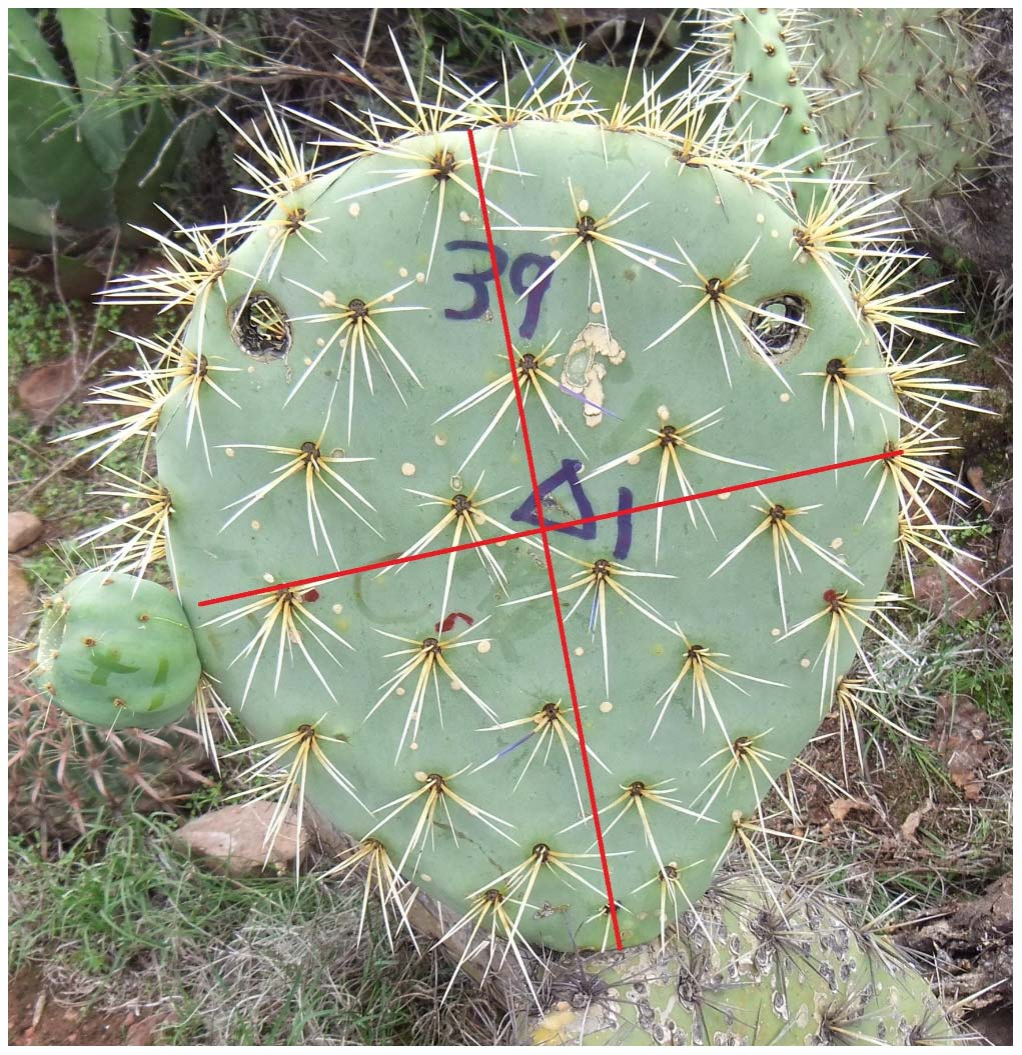
Sampling of the spines and of the total phenolic compounds. We traced an imaginary cross on each cladode, dividing it into four equal sections, and measured the length of each spine (to the nearest 0.5 mm) as well as the number of spines on each areola overlapping the axes. We perforated the mid-section of the arc delimited by the border of the upper quarters of the cladodes, approximately 1 cm away from the border (or close, when an areola obstructed the perforation exactly in this place), using a stainless steel tube with sharpened borders (Ø = 2 cm).

We arbitrarily assigned consecutive numbers to each first-level cladode and then chose three from each plant using a random number table. We traced an imaginary cross on each cladode, dividing it into four equal sections, and measured the length of each spine to the nearest 0.5 mm as well as the number of spines on each areola overlapping the axes (Figure 2). After obtaining the average of both the number of spines on the areolae and their length either i) per plant or ii) per cladode, we compared them for females and hermaphrodites. GLM was applied to compare both physical defense traits of the plants and different states of cladodes: hermaphrodite vs. female, reproductive vs. non-reproductive, and parental vs. non-parental. In the case i), the average value of the physical trait per plant was the unit of comparison and in the case ii), the average value of the physical trait per cladode.

To determine whether spines from both sexual forms have the same physical properties (length, mass), we randomly sampled five spines from five first-level cladodes from the same plant. We measured them to the nearest 0.5 mm, weighed them to the nearest 0.001 g using an analytical balance, and placed them in a drier for two days at 45°C. We compared the intersexual difference in the spine moisture content (fresh – dry biomass) expressed as the proportion of the fresh biomass (transformed with square root to meet homoscedasticity), using GLM. The moisture content is an important trait because some studies have shown that a spine's physical properties improve when the spine is drier [26], [27]. We considered the factor “Plant” nested in the factor “Sex” a random effect and the remaining factors as fixed effects. The factors “Spine length” and “Spine fresh biomass” were considered covariates, transformed with the square root [22], [28].

To sample the defensive substances, we randomly chose three first-level cladodes from each plant belonging to both sexual forms and traced an imaginary cross on the surface of the cladode. We perforated the mid-section of the arc delimited by the border of the upper quarters of the cladodes, approximately 1 cm away from the border, using a stainless steel tube with sharpened borders (Ø = 2 cm; Figure 2). Samples placed in plastic zip lock bags labeled with a permanent marker were transported to the laboratory in a cooler containing freeze gel packs. We used 2 g of the plant tissue to determine TPC content, using the Folin–Ciocalteu method. Tannic acid (TA) was the standard, and an absorbance was measured at 765 nm against a blank sample prepared in an analogue manner, substituting TA with distilled water. We used 80% methanol as a solvent, wrapped the containers with aluminum foil to protect the extract from light, and put them in a refrigerator at −20°C until they were analyzed. We used the calibration curve to convert absorbance to the equivalent of milligrams of TA (eqTA) per gram of fresh biomass of the cladode tissue (mg eqTA g^−1^ FB). We compared both the average TPC concentration per plant and per cladode.

### Reproductive and resource investment trait determination

We harvested each ripened fruit (dark red and soft) from its cladode. If we did not find remnants of the fruits, we considered them as “lost” and did not consider for seed counting fruits partially eaten by frugivores, seed-eating birds, and/or hummingbirds. After measuring the diameter and height of the fruits to the nearest millimeter, we weighed them to the nearest 0.1 g, dried them in an oven at 60°C for three days, and counted the number of seeds in each fruit. We compared the average seed set per fruit, per plant between sexual forms using the K-W [29] test and compared fruit height, diameter, and fresh and dry biomass with GLM (random factor “plant” nested in “sex”).

We counted the total fruit number per plant, per season and the number of fruits eaten or lost at the ripe stage. Applying ANCOVA to compare these traits transformed with natural logarithm between sexual forms, we used the total number of first-level cladodes and/or the total number of fruits per plant as covariates.

To detect possible differences in reproductive allocation patterns between sexes, we compared the relative number of reproductive cladodes on each plant with respect to the number of first-level cladodes. In evaluating possible intersexual differences in vegetative allocation, we compared the number of newly growing cladodes with respect to the number of first-level cladodes, using GLM. The number of first-level cladodes was considered as the covariate in both analyses.

### Estimation of damage caused by vertebrates

We visually estimated the extent of damage exerted by vertebrates to each cladode on each plant, assigned them a category of the cladode volume removed (0%, 1–5%, 6–25%, 26–50%, 51–75%, and >75%), and obtained the weighted average of the proportion of volume removed (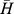) per plant [25], [30]:
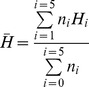
where n_i_ is the number of cladodes in herbivory class *i* and *H*_i_ the midpoint of herbivory class *i*. We obtained values per plant and per cladode level and compared the ln-transformed herbivory levels between sexual forms using one-way ANOVA (plants = repetitions).

## Results

In the study area, we found only female and hermaphrodite forms, confirming female sex in 17 and hermaphrodite in 59 individuals; if not otherwise stated, per plant comparisons were carried out on these plant numbers. During four-year observations, we detected no case of sex change, which is concordant with [10] and [17]. In all cases, when we used GLM (or, Generalized Linear Model (GLZ) with the identity link function), we obtained homoscedasticity and a random distribution of residuals [23].

### Plant architecture and size estimators

The slopes of the relationship between the numbers of cladodes from different levels did not differ between sexes at *P* = 0.5, for all the cases (Table S1; Table S2).

The number of cladodes of the first, second, third, and fourth levels did not differ between sexual forms (Table 1; Table S2).

**Table 1:** Intersexual comparison of the traits analyzed in the study.

Trait analyzed	Type of comparison	n_F_	n_H_	X_F_±CI	X_H_±CI	Statistic	*P*
**Cladode no.**							
1^st^ level	per plant	17	59	24.4±7.4	22.6±4.0	H_1, 76_ = 0.03	*P* = 0.8
2^nd^ level	per plant	17	59	10.2±3.4	9.8±1.9	H_1, 76_ = 0.15	*P* = 0.7
3^rd^ level	per plant	17	59	2.9±1.3	3.7±0.7	H_1, 76_ = 1.48	*P* = 0.2
4^th^ level	per plant	17	59	0.4±0.5	0.8±0.3	H_1, 76_ = 2.17	*P* = 0.14
**Plant size**							
Length [m]	per plant	17	59	2.46±0.48	2.49±0.26	F_1, 74_ = 0.02	*P* = 0.88
Width [m]	per plant	17	59	1.61±0.41	1.66±0.22	F_1, 74 = _0.04	*P* = 0.84
Height [m]	per plant	17	59	0.97±0.10	0.98±0.05	F_1, 74_ = 0.01	*P* = 0.93
**Cladode size**							
Length [cm]	per cladode	414 (17)	1331 (59)	30.1±0.7	31.4±0.3	F_1, 1669_ = 1.69	*P* = 0.19
Width [cm]	per cladode	414 (17)	1331 (59)	25.6±0.5	25.7±0.5	F_1, 1669_ = 0.18	*P* = 0.67
**Spine traits**							
No. per areola	per cladode, per plant	17	59	8.8±1.4	10.8±0.7	F_1, 74 = _6.30	***P*** ** = 0.014**
No. per areola	per cladode, empty & reproductive cladodes	21 (7)	84 (28)	7.9±1.5	10.5±0.8	F_1, 68_ = 7.23	***P*** ** = 0.01**
No. per areola	reproductive cladodes	9 (7)	38 (28)	7.6±2.5	10.6±1.3	F_1, 68 = 6.91_	***P*** ** = 0.01**
Length	per cladode, per plant	17	59	26.2±1.4	25.7±0.6	F_1, 74_ = 0.67	*P* = 0.42
Moisture content [%]*	per cladode	85 (17)	295 (59)	9.4±0.3	8.8±0.2	F_1, 1796_ = 17.20	***P*** **<0.0001**
**Chemical traits**							
TPCs [mg eqTA g-1 FB]	per plant	17	59	7.8±0.7	8.4±0.4	F_1, 74_ = 2.14	*P* = 0.15
TPCs [mg eqTA g-1 FB]	per cladode, empty cladodes	9 (3)	27 (9)	6.7±1.0	8.2±0.6	F_1, 24_ = 6.39	***P*** ** = 0.018**
**Investment traits**							
Seed output	per fruit, per plant	7	27	178.7±50.3	248.4±32.7	H_1, 35_ = 5.6	***P*** ** = 0.018**
Seed output**	per fruit, per plant	7	27	207.6±31.1	260.9±38.4	H_1, 26_ = 4.3	***P*** ** = 0.04**
Relative reproductive cladode frequency	per plant	17	59	2.8±1.2	2.0±0.66	F_1, 73_ = 0.8	*P* = 0.36
Relative daughter cladode frequency	per plant	17	59	0.8±0.6	1.1±0.33	F_1, 73_ = 0.6	*P* = 0.44
**Damage** ***							
1^st^ level	per plant	17	59	3.1±1.1	3.4±0.6	F_1, 74_ = 0.38	*P* = 0.54
2^nd^ level	per plant	17	59	3.7±1.9	3.1±1.1	F_1, 74_ = 0.19	*P* = 0.66
3^rd^ level	per plant	15	53	2.8±2.8	3.1±1.5	F_1, 66_ = 0.06	*P* = 0.81
Total	per plant	17	59	4.1±1.1	3.5±0.6	F_1, 74_ = 0.19	*P* = 0.66

n_F_ and n_H_ - sample sizes for females and hermaphrodites (individuals or cladodes), respectively. “Cladode no.” – the number of cladodes per branching level. “Cladode size” concerns the 1^st^-level cladodes. The numbers of cladodes per branching level per plant were compared using K-W, and other variables using GLM or ANOVA. In all GLM tests when cladode was a unit of comparison, the variable “plant” was nested in the variable “sex.” The number of individuals of each sex in the nested comparisons is in parentheses after the number of cladodes. Total phenolic compounds (TPCs) are given in equivalents of tannic acid (TA) per fresh biomass (FB) [mg eqTA g^−1^ FB]. Significant *P* values are in bold.

*– means and CI for non-transformed data; significance test for square-root–transformed data;

**– plants bearing more than one fruit;

***– means and CI for non-transformed data; significance test for ln-transformed data.

We did not find intersexual differences in maximum plant length, maximum plant width, or maximum plant height (Table 1; Table S3). First-level cladodes did not differ in length and width between females and hermaphrodites (Table 1; Table S4).

### State-dependent physical and chemical defense traits

Females had on average two spines fewer per areola, per plant than hermaphrodites (8.8±1.4 vs. 10.8±0.7; Table 1; Table S5). We treated the variable “plant” as a repetition inside the variable “sex,” and each repetition was the average number of spines per areola, per plant.

We did not find significant differences in physical defense traits either between sexual forms or between parental states when we compared 1) cladodes of the plants that bore either only empty cladodes or 2) empty together with parental cladodes. We found significant intersexual differences when we compared 21 cladodes from 7 female plants to 84 cladodes from 28 hermaphrodite plants bearing empty and reproductive cladodes: Areolae on hermaphrodite reproductive cladodes had on average 2.65 spines more than on female reproductive cladodes (10.5±0.78 vs. 7.85±1.54; Table 1). Furthermore, a planned comparison between reproductive female and hermaphrodite showed that the latter bore 3.3 more spines per areola (7.6±2.5 vs. 10.6±1.3; Table 1). The number of areolae per cladode did not differ significantly between either sexual form or reproductive state (Table S6).

We did not find intersexual differences in the average spine length per plant (26.2±1.37 vs. 25.7±0.6; Table 1; Table S7). Similarly, we did not find significant intersexual differences in the average spine length per cladode when we compared the groups of plants with either empty cladodes (E) or empty and parental cladodes (PE). In the RE group, (empty and reproductive cladodes), we found no effect of the reproductive state on this trait. However, the presence of reproductive cladodes increased the difference between sexual forms: female cladodes in both non-reproductive and reproductive states had longer spines than hermaphrodite ones, even when this difference was not significant (*F*_1, 68_, *P* = 0.32). This intersexual difference was less obvious in plants with empty cladodes and absent in plants with empty and parental cladodes (Table S6).

The distribution of the content of spine moisture was right-skewed for both sexual forms. Even when GLM showed no significant differences between sexes (*F*_1, 1796_ = 0.84, *P* = 0.36), the Bonferroni *post hoc* test and the comparison of confidence intervals (CI) did. The lack of significance may have occurred due to a considerable variance in plant(sex) (*F*_73, 1796_ = 20.36, *P*<0.0001). For this reason, we performed a planned comparison between sexes and found that female spines lost more moisture than hermaphrodite spines (9.4±0.3 vs. 8.8±0.2; Table 1). Both covariates had a significant effect on moisture content (*F*_1, 1796_ = 38.17, *F*_1, 1796_ = 62.52, for spine length and spine fresh biomass, respectively; *P*<0.0001 for both). Twenty-five female and two hermaphrodite spines were lost during field and lab work (Table S8).

When we compared the average TPCs per plant with GLM, we found no intersexual differences (7.8±0.7 vs. 8.4±0.4; Table 1; Table S9). A planned comparison between sexual forms within group E (empty cladodes) with the variable “plant” nested in the variable “sex” (fixed effect) showed that hermaphrodite cladodes contained a higher concentration of TPCs than female cladodes by 1.48 mg eqTA g^−1^ FB (22.1%) (8.2±0.6 vs. 6.7±1.04; *F*_1, 24_ = 6.39, *P* = 0.018; Table 1; Table S10).

**Figure 3:**
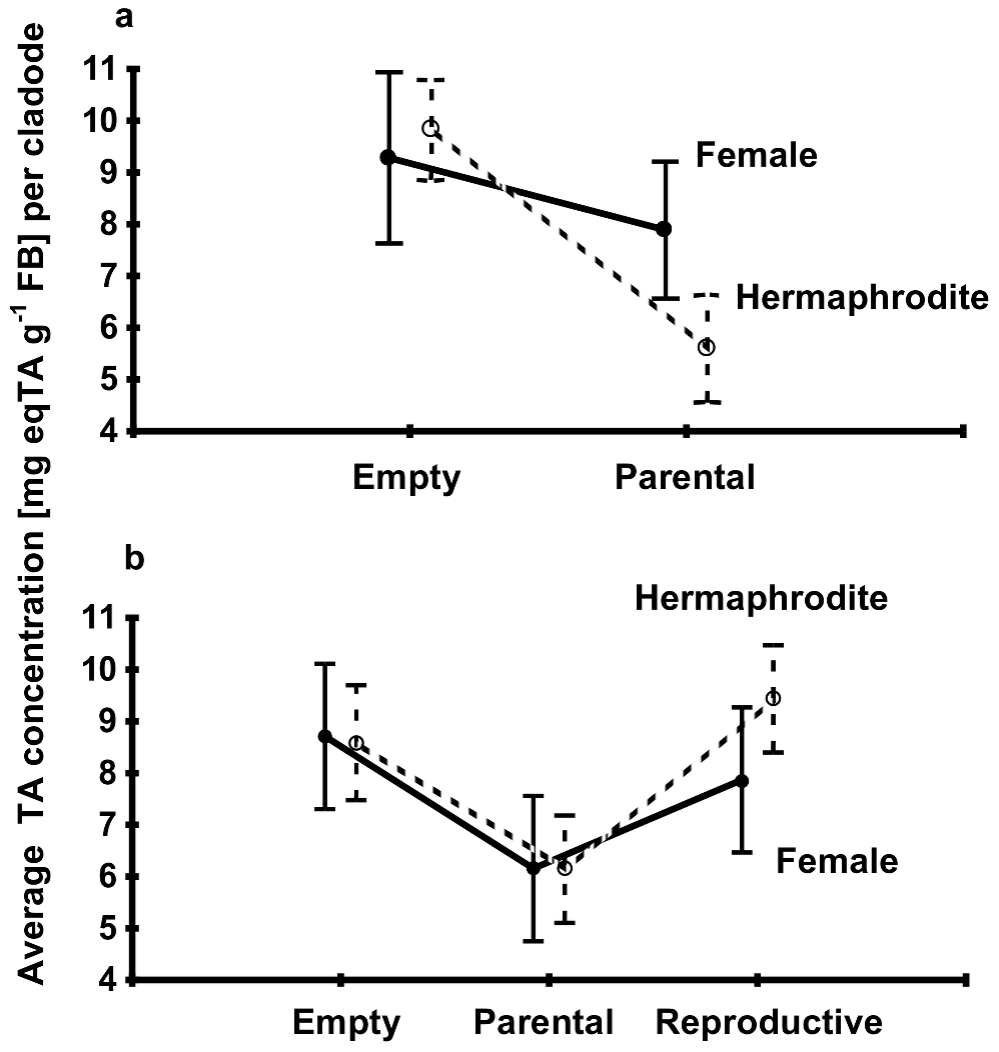
State-dependent comparison of the average total phenolic compounds (TPCs) per cladode in terms of tannic acid (TA), in 1^st^-level cladodes. a) Comparison of empty (non-parental) and parental, female, and hermaphrodite cladodes in the group of plants that bore empty and parental cladodes. Empty cladodes contained a higher TPC concentration than parental ones. Parental female cladodes contained a higher TPC concentration than parental hermaphrodites (significant Bonferroni test at 0.05). b) Comparison of the concentration of TPCs from empty, parental, and reproductive cladodes in the group of plants that bore each cladode from a different state. Parental hermaphrodite cladodes contained a lower TPC concentration than empty and reproductive cladodes.

The comparison of the parental state of the cladodes within the group PE (parental and empty cladodes) confirmed that hermaphrodite parental cladodes contained a significantly lower concentration of TPCs than non-parental ones. The same tendency observed in female cladodes was not significant. Also, female non-parental cladodes contained a significantly higher concentration of TPCs than the hermaphrodite parental cladodes, and female parental cladodes contained 2.3 mg eqTA g^−1^ FB (41%) greater concentration of TPCs than hermaphrodites (Figure 3a; Table 2). The comparison of the plants from the group RPE (one cladode from each state) confirmed the prediction derived from plants in other groups: Parental cladodes had a lower concentration of TPCs than empty and reproductive cladodes. This difference was more obvious in hermaphrodite plants (Figure 3b; Table 3). In the group of plants bearing reproductive and empty cladodes, there was no significant effect of any factor (there were only six female cladodes nested in two plants; Table S10).

**Table 2:** Sex-dependent average tannic acid (TA) concentration [mg eqTA g^−1^ FB] per cladode.

Factor	SS	df	MS	F	*P*
Parental status	59.53	1	59.53	19.22	0.0002
Parental status ^x^ Sex	15.16	1	15.16	4.90	0.036
Sex	6.80	1	6.80	1.21	0.289
Plant (Sex)	73.96	13	5.69	1.84	0.093
Error	77.41	25	3.10		

The factor “Plant” nested in the factor “Sex” was considered a random effect and the remaining factors as fixed effects. GLM results for plants bearing parental and empty cladodes. Female parental cladodes contained a higher concentration than hermaphrodites by 2.3 mg eqTA g^−1^ FB (41%).

**Table 3:** Sex-dependent average tannic acid (TA) concentration [mg eqTA g^−1^ FB] per cladode.

Factor	SS	df	MS	F	*P*
Cladode type	64.24	2	32.12	11.37	0.0002
Cladode type ^x^ Sex	6.85	2	3.43	1.21	0.31
Sex	2.61	1	2.61	0.93	0.35
Plant (Sex)	42.38	15	2.83	1.00	0.48
Error	81.95	29	2.83		

The factor “Plant” nested in the factor “Sex” was considered a random effect and the remaining factors as fixed effects. GLM results for plants bearing empty, parental, and reproductive cladodes (cladode type). Significant differences occurred among empty and parental cladodes of both sexes and among all types of hermaphrodite cladodes.

### Relationship between defense traits

**Figure 4:**
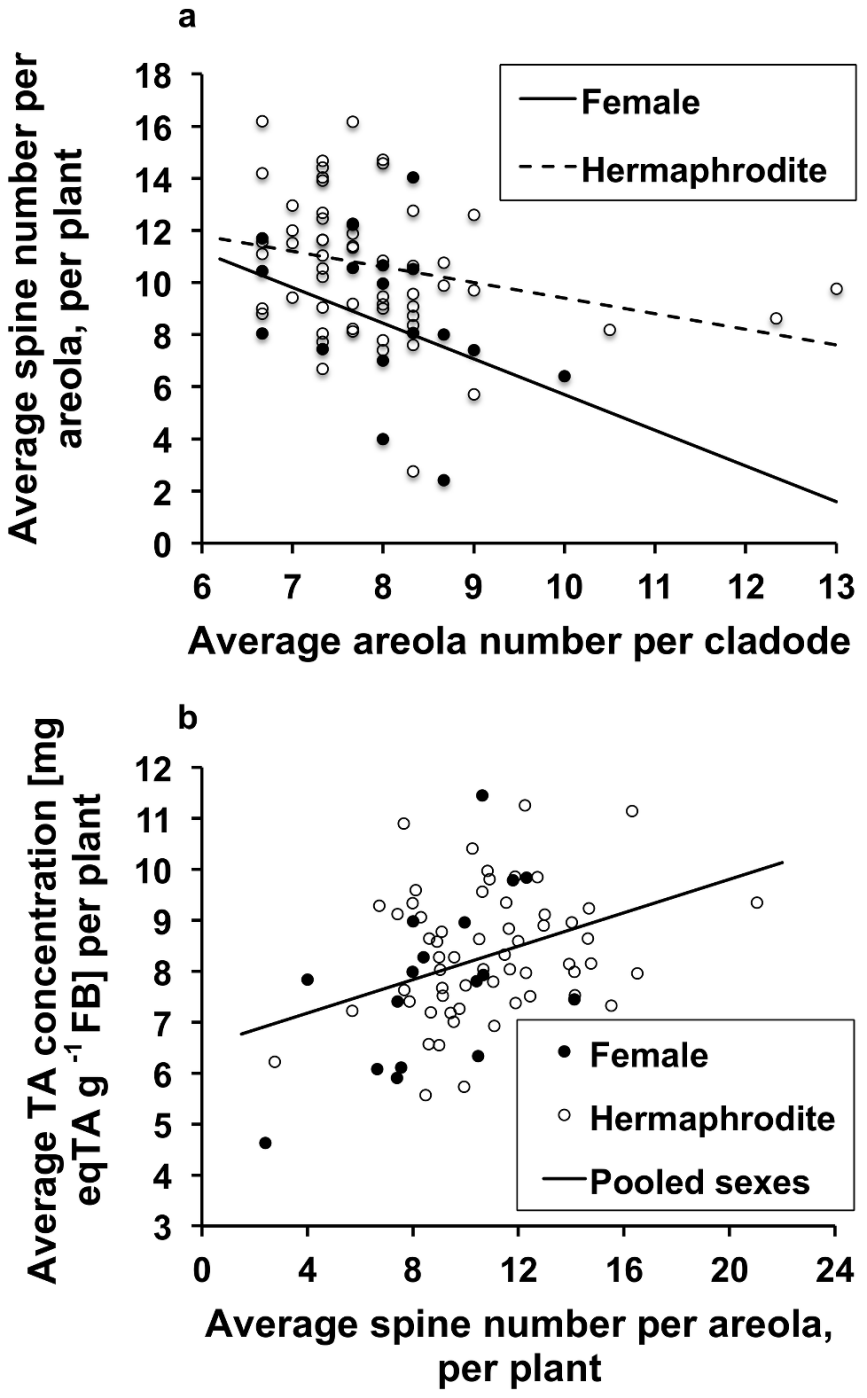
Relationship between defense traits. a) Comparison of the regression lines between average areolae number on a cladode (X) and average spine number per areola, per plant (Y): Y = 19.43 – 1.38 X, and Y = 15.43 – 0.6 X, for females and hermaphrodites, respectively. The effect of average areola number was significant (*F*_1, 3_ = 8.33, *P* = 0.005). The slopes were not significantly different between sexes (F_1, 3_ = 1.1, *P* = 0.29), contrary to the intercepts (F_1, 3_ = 10.5, *P* = 0.002). The adjustment of the model was significant (*F*_3, 71_ = 6.66, *P* = 0.0005). b) Average spine number per plant, per areola (X), and average TPCs in terms of tannic acid concentration (Y) per plant. There was no significant intersexual difference between either slopes (*F*_1, 3_ = 3.11, *P* = 0.08) or intercepts (*F*_1, 3_ = 0.42, *P* = 0.52) for this relationship. The relationship for the pooled data was Y = 6.52+0.164 X (*F*_1, 74_ = 11.87, *P* = 0.0009; adj. *r* ^2^ = 12.66%).

There was a significant negative but weak relationship between the average spine number per plant (ASNPP) in an areola and the average areolae number per plant; the slopes were not significantly different between sexes, contrary to the intercepts (AANPP; Figure 4a). We removed from the analysis four outliers (>2 SD from the regression, using studentized residuals; one for females and three for hermaphrodites; Table S11). We found a positive relationship between the average TPC concentration per plant and average spine number in an areola per plant (Figure 4b; Table S12).

### Intersexual difference in reproductive and resource investment traits

In 2011, 10 female and 38 hermaphrodite individuals blossomed whereas in 2010, 13 female and 45 hermaphrodite plants did so; 7 female and 21 hermaphrodite individuals did not repeat reproduction in 2011. Four female and fourteen hermaphrodite individuals that did not reproduce in 2010 reproduced in 2011, and six female and twenty-four hermaphrodite individuals repeated reproduction in both seasons (Table S13). The remaining 28 individuals never reproduced during four years of observation (2009–2012) [23].

**Figure 5:**
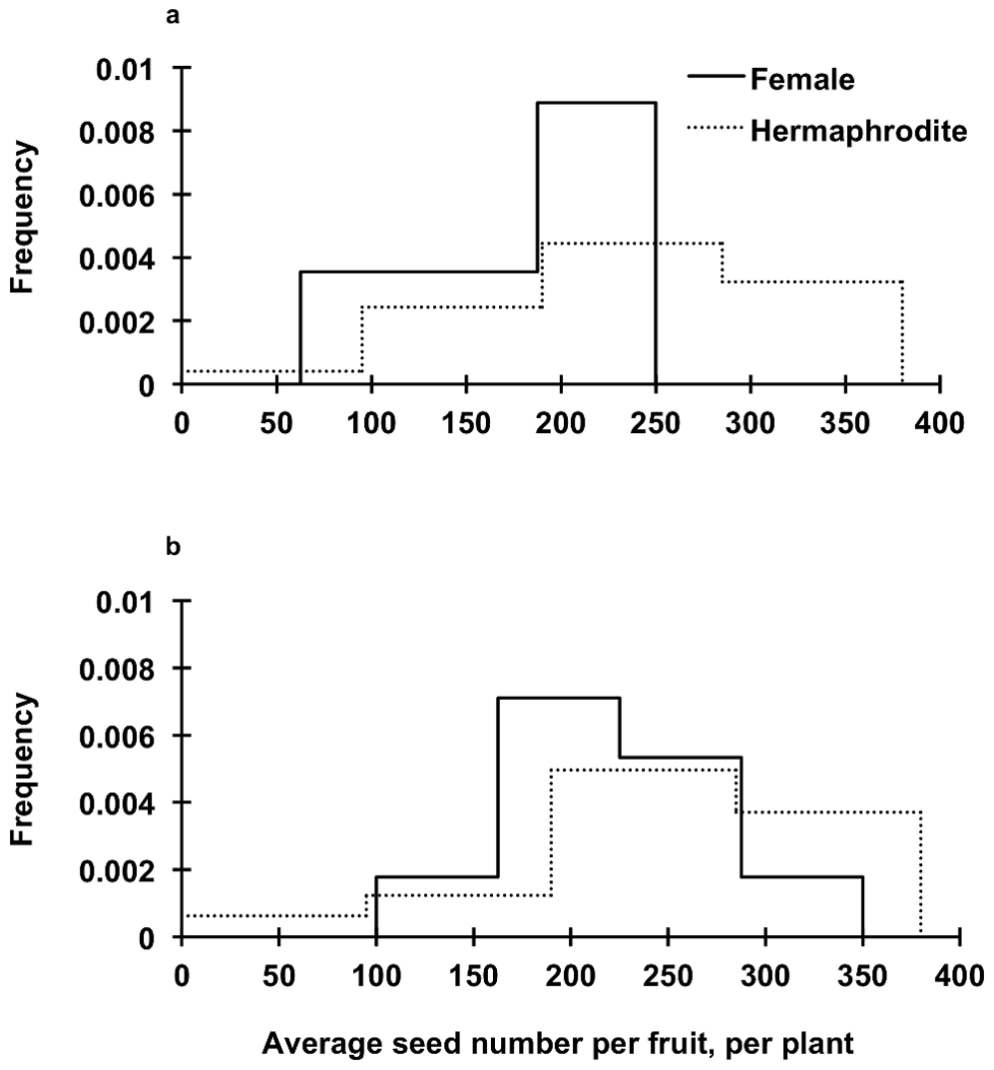
Intersexual differences in reproductive output. a) estimated as seed number per fruit for all the plants sampled (*P* = 0.04). b) With exclusion of plants bearing only one fruit on one cladode (*P* = 0.02).

We obtained 33 and 127 ripened fruits from 9 female and 27 hermaphrodite plants respectively, before they were either eaten or lost. We found no significant intersexual difference in the average seed set per plant when we compared seeds from all the fruits (218.1±101.9 vs. 248.4±32.7, H_1, 36_ = 2.89, *P* = 0.089, for females and hermaphrodites, respectively). However, when we removed from the analysis one outlier (4.16 SD from the mean, using studentized residuals) corresponding to a female plant that bore only one fruit with 538 seeds, female fruits contained on average 59 seeds fewer than the hermaphrodite fruits (178.1±50.3 vs. 248.4±32.7; Figure 5a). This outlier represented the only female fruit of this size (80×68 mm, for length and diameter, respectively). To exclude the effect of the “atypical” plants that bore only one fruit, we compared fruits from those plants that bore more fruits (only one female and three hermaphrodite plants were removed): Female fruits contained on average 53 seeds fewer than hermaphrodites (207.6±31.1 vs. 260.9±38.4; Figure 5b; Table 1; Table S14).

We found no intersexual differences in any estimator of fruit size: height (*F*_1, 159_ = 0.0001, *P* = 0.99), diameter (*F*_1, 159_ = 0.007, *P* = 0.94), fresh biomass (*F*_1, 159_ = 0.40, *P* = 0.53), or dry biomass (*F*_1, 148_ = 0.34, *P* = 0.56; 12 fruits were lost during the lab work; Table S15).

Plants from both sexes did not differ significantly in average fruit number produced during season per plant (AFN; comprises either dead before ripening and ripe fruits; ANCOVA *F*_1, 57_ = 0.91, *P* = 0.35; first-level cladode number (CN) as covariate). Neither differed in the number of ripe fruits either eaten or lost (*F*_1, 44_ = 1.03, *P* = 0.32; CN and AFN as covariates; Table S16).

We identified no intersexual differences in the relative investment in reproductive and vegetative structures. There was no difference either in the number of reproductive cladodes (2.8±1.2 vs. 2.0±0.66; Table S17) or in the number of daughter cladodes (0.8±0.6 vs. 1.1±0.33; Table 1; Table S18) with respect to the number of first-level cladodes.

### Estimation of damage caused by vertebrates

The sexual forms did not differ either in overall damage level or in damage estimated separately per cladode level (Table 1; Table S19). We did not estimate damage for the fourth-level cladodes (only two plants had visible damage).

## Discussion

### Herbivory and defense

We showed that spines of both sexual forms examined here differ in moisture content. Malainine et al. [27] found that most of the dry biomass of *O. ficus-indica* spines was composed of cellulose (47.9%) and other polysaccharides (48.4%) and that only 1.2% accounted for potentially evaporable fat and wax. In *O. ficus-indica*, the spine tensile modulus and the bending strength decrease when the moisture content increases [26], [27]. The latter two studies suggested that hermaphrodite spines are more resistant to physical pressure than female spines not only because they contain less water but also because their tissue is more compacted.

Hermaphrodite reproductive cladodes are spinier than female cladodes; that is, reproduction reinforces the production of spines more in the more costly sex. Obviously, it is difficult to imagine that a plant allocates resources differentially to defense by reabsorbing the existing spines from the non-reproductive cladodes; rather, a plant invests more energy in the production of new spines when a cladode turns reproductive. We did not detect such differential allocation among tissues in the case of the TPCs, probably because, according to ODT, these substances are energetically cheap [31]. We found intersexual differences in TPCs when we compared empty cladodes: Hermaphrodite cladodes contained a higher concentration of TPCs. Both differences in physical and chemical defenses between sexual forms and a probable effect of the cladode's reproductive state on spine density would confirm the predictions of ODT if we had confirmed either intersexual or intertissular differences in costs. Differences in energetic expenditures between reproductive and non-reproductive cladodes are rather obvious; however, the only reproductive trait that differed between sexual forms was the seed output per plant and its variance, which were both higher in hermaphrodites.

Goats, common in the study zone, are known to be generalist herbivores, and spines of both sexual forms of *O. robusta* are not a totally effective defense against them. This factor is probably why we did not find intersexual differences in damage level to cladode biomass.

Pimienta-Barrios et al. [32] found in *O. ficus-indica* a lower concentration of TPCs in cladodes bearing either eight or more daughter cladodes and explained it as a result of competition for resources between parent and daughter cladodes [33]. We obtained a similar result in that almost all of the parental first-level cladodes produced only one daughter. We may have found evidence to suggest the existence of a cost of chemical defense: If such a cost had not existed, parental and daughter cladodes would have contained the same concentrations of TPCs. The ultimate explanation may rely on the following arguments: 1) New cladodes do not have physical defenses because spines require time to grow; therefore, 2) they should be defended by defensive substances. 3) The main source of defensive substances and/or carbon compounds that can be converted to defensive substances at their early growing stage is the mother cladode, and 4) the loss of new cladodes decreases fitness because first-level cladodes can turn reproductive in the future. If the only cause of the lower TPC concentration in mother cladodes had been the competition for resources, the reproductive cladodes also would have contained a lower concentration. Regardless of the explanation, a lower concentration of TPCs in mother cladodes seems to be a trade-off between different physiological processes, between the investment in the growth of old and new vegetative biomass, between defense of old and new cladodes, or between defense of parental and growth of the newly growing cladode.

### Relationship between defense traits

A lower average spine number per areola on cladodes with a higher number of areolae is probably an outcome of a trade-off between these two traits: The regression line for the realistic values of the average areolae number in hermaphrodites lies above the regression line for females because the spine number per areola is higher in the former (Figure 4). In other *Opuntia* species, the final areolae number occurs within 2–4 weeks after cladode initiation [34]. The negative correlation between two physical defense traits is explained by the fact that the same pool of energy and thus metabolites may be used to construct many areolae with fewer spines and fewer areolae with more spines; both solutions may work equally well against vertebrate herbivores. A plant with a low density of areolae cannot increase their number because of the ontogenetic constraint, so to achieve a deterrent effect, it should increase spine number. A plant with a higher density of areolae may achieve the same deterrent effect with fewer spines per areolae. A positive correlation between physical and chemical defense traits is probably an outcome of the fact that they aid the plant in defense against different herbivores. Vertebrates are deterred by both phenolic compounds and spines whereas invertebrates are deterred only by the former; an increase in spinescence and a decrease in the concentration of phenolic compounds when both types of herbivores are present is not an optimal solution from the ODT perspective.

The production of both kinds of defense seems not to be problematic for the plant. Because the biosynthetic pathways of each differ, they do not compete directly for the same pool of energy. Additionally, phenolic compounds (mainly tannins) are energetically cheap [31]. Koricheva et al. [15] found a positive but non-significant correlation between physical and chemical traits at the inter-specific level. They concluded that the negative correlations between defense traits are not a general outcome of the plant defense, with one exception: constitutive vs. induced defenses. A positive correlation was confirmed only for compounds linked by either biosynthetic reactions or by genes. We found here in one plant species two examples of a relationship between defense traits: One of them is probably a trade-off and another a positive relationship that can be explained from the ODT perspective as an optimal solution when either kind of defense deters different types of herbivores [25].

### Herbivory and sexual polymorphism

We found no differences in direct reproductive traits that could be easily linked to high-energy expenditures. It seems that besides the seed output per plant, both sexual forms did not differ in their allocation to reproduction. A question then arises: Is a higher seed output in hermaphrodites a sufficient selective advantage to pay off such high expenditures to physical defense? If this is true, why then do females persist in this population if they have the two apparent disadvantages of a lower seed output and a lower protection against herbivores? The hypothesis concerning the ecological cost of defense is a possible explanation because only 40% of hermaphrodites and 35% of females that reproduced in 2010 repeated reproduction in 2011. In 2011, 58% of females and 64% of hermaphrodites reproduced; in 2010, 76% of both sexes did so. These seasonal fluctuations in reproductive output may possibly change the defense strategy from season to season; a sexual form that outcompetes another in one season can be outcompeted in the future, so the lifespan reproductive success of both sexes can be similar.

### Possible sources of intersexual differences in reproductive cost

Additional evidence of a higher reproductive cost of hermaphrodites of *O. robusta* is a larger periant diameter (that probably implies higher flower biomass) and a higher nectar content than in female flowers [10], [11]. In a trioecious population of *O. robusta*, one group [19] found that hermaphrodites of this species have a higher reproductive output than females and produce more fruits per plant. These results together with ours seem to contradict some very well documented case studies [35]. We believe that the higher reproductive cost of female sexual forms should not be generalized because meta-analysis studies frequently show a considerable variance [6], [24], [36].

In this investigation, we detected no intersexual differences in vegetative body estimators attributable to differences in reproductive cost. A long-term study is necessary to confirm whether a higher physiological cost of defense and reproduction of one sexual form is traded off by a lower survival and a lower actual reproductive output in a less defended sexual form is thus compensated by a greater lifetime reproductive output.

## Conclusions

In this study, we showed that the seed output is higher in hermaphrodites than in females of *O. robusta* but also has a higher variance. Furthermore, the hermaphrodite sexual form is on average spinier than the female form, and reproductive cladodes are spinier in hermaphrodites than in females. In addition, we showed that hermaphrodite empty cladodes contain a higher concentration of phenolic compounds than female empty cladodes and found a possible trade-off between the production of phenolic compounds in parental cladodes and their content in daughter cladodes. There also was a possible trade-off between the density of spines on areolae and average number of areolae per cladode, and a positive relationship between physical and chemical defense traits.

With the current data, we cannot conclude that sexual polymorphism in the study population is herbivore mediated. In addition, we cannot make inferences about the possible evolution from gynodioecy to dioecy in *O. robusta* because of the need for accurate information concerning the costs of inbreeding, the entire costs of reproduction, the lifetime reproductive success, the estimation of vegetative growth traits, and possible competition for pollinators with other plant species.

## Supporting Information

Figure S1 **Hermaphrodite and female fruits.**
(TIF)

Table S1 **Relationship among the number of cladodes from different levels, for female and hermaphrodite individuals.**
(PDF)

Table S2 **Number of cladodes from the first, second, third and fourth levels.**
(XLSX)

Table S3 **Length, width and height of all plants.**
(XLSX)

Table S4 **First-level cladode size (length and width) per plant.**
(XLSX)

Table S5 **Average spine physical traits per plant.**
(XLSX)

Table S6 **Average spine physical traits per cladode.**
(XLSX)

Table S7 **Average spine length per plant.**
(XLSX)

Table S8 **Spine moisture content.**
(XLSX)

Table S9 **Average total phenolic compound concentration per plant.**
(XLSX)

Table S10 **Average total phenolic compound concentration per cladode.**
(XLSX)

Table S11 **Average spine number in an areola per plant vs. average areolae number per plant.**
(XLSX)

Table S12 **Average total phenolic compound concentration per plant vs. average spine number in an areola per plant.**
(XLSX)

Table S13 **Plant blossoming per season.**
(XLSX)

Table S14 **Seed number per fruit per plant.**
(XLSX)

Table S15 **Average fruit height, diameter, and fresh and dry biomass.**
(XLSX)

Table S16 **Fruit set per plant per season.**
(XLSX)

Table S17 **Relative number of reproductive cladodes per plant.**
(XLSX)

Table S18 **Relative number of sprouts per plant.**
(XLSX)

Table S19 **Damage estimate per plant per all cladodes and per cladode levels.**
(XLSX)

File S1 **Basic information concerning** ***Opuntia robusta.***
(PDF)
