# Supplementary figures and images for: Chemical and Physical Defense Traits in Two Sexual Forms of Opuntia robusta in Central Eastern Mexico
Source: PLoS One. 2014 Mar 5;9(3):e89535. doi: 10.1371/journal.pone.0089535 (PMC3943789; doi:10.1371/journal.pone.0089535)

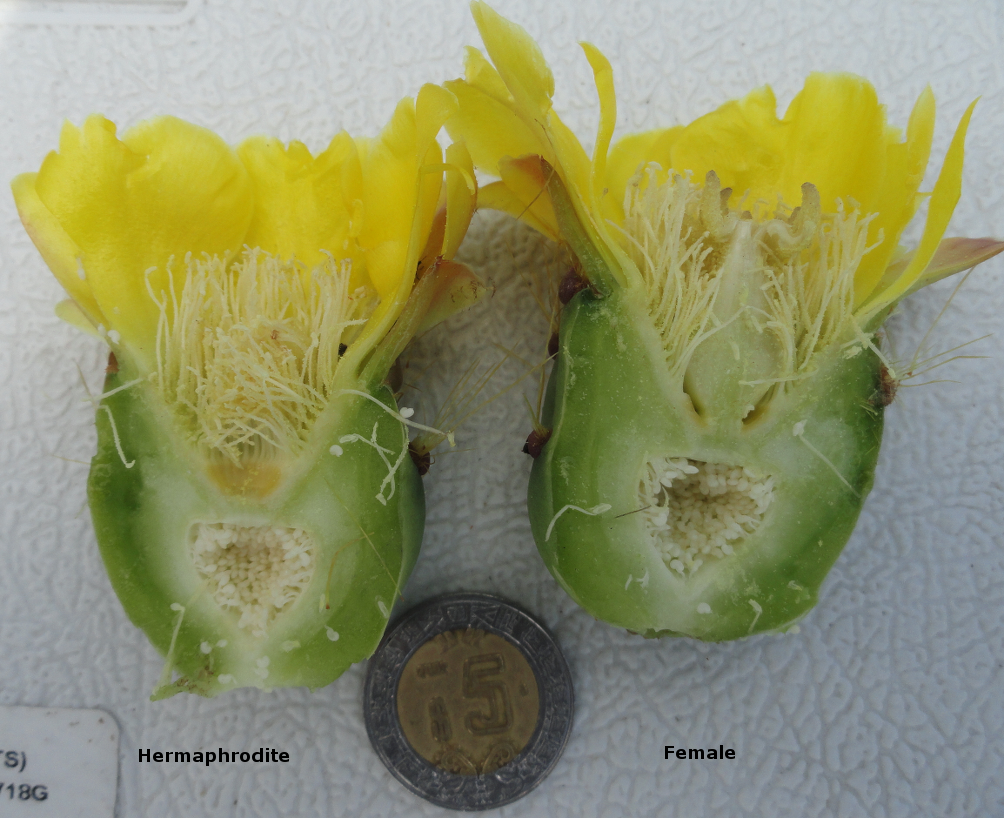

Supplement: Figure S1 — Hermaphrodite and female fruits. (TIF) [file pone.0089535.s001.tif]
