# Supplementary material for: Chemical and Physical Defense Traits in Two Sexual Forms of Opuntia robusta in Central Eastern Mexico
Source: PLoS One. 2014 Mar 5;9(3):e89535. doi: 10.1371/journal.pone.0089535 (PMC3943789; doi:10.1371/journal.pone.0089535)
Supplement: Table S1 — Relationship among the number of cladodes from different levels, for female and hermaphrodite individuals. (PDF) [file pone.0089535.s002.pdf]

Table S1. Relationship among the number of cladodes from different levels, for female and hermaphrodite individuals. Critical values, significance, and coefficients of determination of the ordinary linear regression for the intersexual slope comparison of the relationship between the number of cladodes from different levels (df = 1 and df = 3). Cladode numbers were transformed using the natural logarithm to meet the linearity and continuity of the data.

**Cladode level**	**2nd**	**3rd**	**4th**
**1st**	$F = 1.7$ $P = 0.2$, $r^2 = 0.85$	$F = 0.12$, $P = 0.73$, $r^2 = 0.55$	$F = 0.08$, $P = 0.07$, $r^2 = 0.77$
**2nd**	–	$F = 0.59$, $P = 0.44$, $r^2 = 0.58$	$F = 0.25$, $P = 0.62$, $r^2 = 0.42$
**3rd**	–	–	$F = 0.17$, $P = 0.69$, $r^2 = 0.34$
