# Supplementary material for: Chemical and Physical Defense Traits in Two Sexual Forms of Opuntia robusta in Central Eastern Mexico
Source: PLoS One. 2014 Mar 5;9(3):e89535. doi: 10.1371/journal.pone.0089535 (PMC3943789; doi:10.1371/journal.pone.0089535)
Supplement: File S1 — Basic information concerning Opuntia robusta. (PDF) [file pone.0089535.s021.pdf]

Basic information concerning *Opuntia robusta*

The species belongs to platyopuntias that is, *Opuntias* which develop flattened photosynthesizing stems and shoots called cladodes. The cladodes are covered by spines (vestigial leaves) that grow on highly specialized branches: these branches are called areolae [1]. Parenchyma of *Opuntia* species contain phenolic compounds such as quercetin, kaempferol, and isorhamnetin. These flavonoids have been also reported in *O. robusta* [2]. The presence of ferulic, gallic and coumaric acids was reported by [3]. Virtually nothing is known about the defensive functions of these substances in this species. Its fruits contain phenolics (including flavonoids) and betalains [4]. Spines also contain flavonoids, however, their function is not clear [2]. The plant scars its injuries by producing a viscous resin that stiffens.

Most of the floral sprouts appear in March. The blossoming period spans from May through June. We found the first fruiting plants in April and the last in November [5].

References

1. Muñoz-Urias A, Palomino-Hasbach G, Terrazas T, García-Velázquez A, Pimienta-Barrios E (2008) Variación anatómica y morfológica en especies y entre poblaciones de *Opuntia* en la porción sur del Desierto Chihuahuense. Boletín de la Sociedad Botánica de México: 1-11.
2. Stintzing FC, Carle R (2005) Cactus stems (*Opuntia* spp.): a review on their chemistry, technology, and uses. Mol Nutr Food Res 49: 175-194.

3. Guevara-Figueroa T, Jimenez-Islas H, Reyes-Escogido ML, Mortensen AG, Laursen BB, et al. (2010) Proximate composition, phenolic acids, and flavonoids characterization of commercial and wild nopal (*Opuntia* spp.). J Food Compos Anal 23: 525-532.

4. Chavez-Santoscoy RA, Gutierrez-Uribe JA, Serna-Saldivar SO (2009) Phenolic composition, antioxidant capacity and in vitro cancer cell cytotoxicity of nine prickly pear (*Opuntia* spp.) juices. Plant Foods Hum Nutr 64: 146-152.

5. Rodríguez-Zapata O (1981) Fenología reproductiva y aporte de frutos y semillas en dos nopaleras del Altiplano Potosino-Zacatecano. Monterrey, Nuevo León, México.: Universidad Autónoma de Nuevo León. 158 p.
